# Comparison of weighting approaches for genetic risk scores in gene-environment interaction studies

**DOI:** 10.1186/s12863-017-0586-3

**Published:** 2017-12-16

**Authors:** Anke Hüls, Ursula Krämer, Christopher Carlsten, Tamara Schikowski, Katja Ickstadt, Holger Schwender

**Affiliations:** 1 IUF-Leibniz Research Institute for Environmental Medicine, Düsseldorf, Germany; 2 Faculty of Statistics, TU Dortmund University, Dortmund, Germany; 3 Department of Medicine, University of British Columbia, Vancouver, BC Canada; 4 Institute for Heart and Lung Health, Vancouver, BC Canada; 5 School of Population and Public Health, University of British Columbia, Vancouver, BC Canada; 6 Mathematical Institute, Heinrich Heine University, Düsseldorf, Germany

**Keywords:** Polygenic approach, Training dataset, Internal weights, External weights, Simulation study, Power, Type I error

## Abstract

**Background:**

Weighted genetic risk scores (GRS), defined as weighted sums of risk alleles of single nucleotide polymorphisms (SNPs), are statistically powerful for detection gene-environment (GxE) interactions. To assign weights, the gold standard is to use external weights from an independent study. However, appropriate external weights are not always available. In such situations and in the presence of predominant marginal genetic effects, we have shown in a previous study that GRS with internal weights from marginal genetic effects (“GRS-marginal-internal”) are a powerful and reliable alternative to single SNP approaches or the use of unweighted GRS. However, this approach might not be appropriate for detecting predominant interactions, i.e. interactions showing an effect stronger than the marginal genetic effect.

**Methods:**

In this paper, we present a weighting approach for such predominant interactions (“GRS-interaction-training”) in which parts of the data are used to estimate the weights from the interaction terms and the remaining data are used to determine the GRS. We conducted a simulation study for the detection of GxE interactions in which we evaluated power, type I error and sign-misspecification. We compared this new weighting approach to the GRS-marginal-internal approach and to GRS with external weights.

**Results:**

Our simulation study showed that in the absence of external weights and with predominant interaction effects, the highest power was reached with the GRS-interaction-training approach. If marginal genetic effects were predominant, the GRS-marginal-internal approach was more appropriate. Furthermore, the power to detect interactions reached by the GRS-interaction-training approach was only slightly lower than the power achieved by GRS with external weights. The power of the GRS-interaction-training approach was confirmed in a real data application to the Traffic, Asthma and Genetics (TAG) Study (*N* = 4465 observations).

**Conclusion:**

When appropriate external weights are unavailable, we recommend to use internal weights from the study population itself to construct weighted GRS for GxE interaction studies. If the SNPs were chosen because a strong marginal genetic effect was hypothesized, GRS-marginal-internal should be used. If the SNPs were chosen because of their collective impact on the biological mechanisms mediating the environmental effect (hypothesis of predominant interactions) GRS-interaction-training should be applied.

**Electronic supplementary material:**

The online version of this article (10.1186/s12863-017-0586-3) contains supplementary material, which is available to authorized users.

## Background

For many diseases, genetic influences are exceedingly complex and cannot be explained by simple Mendelian modes of inheritance only. Moreover, genetic and environmental factors may jointly contribute to susceptibility clarifying the importance of analyzing gene-environment (GxE) interactions, which can be defined as “a different effect of environmental exposure in disease risk in persons with different genotypes” [1].

Since most complex diseases are influenced by hundreds of genetic variants each having a small effect on its own, polygenic approaches that deal with the genetic basis *en masse* often access more of the heritable component of complex traits than is possible by single-variant approaches [2]. The most common polygenic approach is the weighted genetic risk score (GRS) approach in which a weighted GRS is calculated from a pre-selected number of genetic variants to define a person’s individual genetic risk for disease development [3].

One of the first GRS applications was published by Purcell et al. who used GRS to argue that schizophrenia has a polygenic risk [4]. Although their genome-wide association study (GWAS) identified few individually significant single nucleotide polymorphisms (SNPs), they provided evidence for a substantial polygenic component to risk of schizophrenia involving thousands of common alleles of very small effect. In addition, GRS show promise for patient stratification and subphenotyping [2]. Hamshere et al. showed that among bipolar disorder cases GRS for schizophrenia risk could distinguish schizo-affective cases from others [5]. Moreover, GRS were successfully used in interaction analyses to examine the genetic susceptibility to air pollution-induced type 2 diabetes [6], air pollution-induced airway inflammation [7] and fried food-induced obesity [8].

The high power of GRS approaches to detect GxE interactions has been confirmed in a recent methodological paper by Aschard [9]. In this publication, Aschard showed that if most interaction effects point into the same direction, the use of GRS increases the power to detect GxE interactions in comparison to the common univariate single-variant approaches, e.g. with Bonferroni correction, and the joint test of main genetic and interaction effects [9, 10]. Furthermore, by combining SNPs of a certain biological pathway, GRS can be used as a simple statistical approach for the complex biological pathways through which environment-induced diseases might be caused [7].

GRS have been employed to summarize genetic effects among an ensemble of markers that do not individually achieve significance and to estimate the variance explained by a marker panel [3]. In these applications, the gold standard is to use external weights, e.g. marginal genetic effects estimated in an independent study population [3, 11].

In a recent publication, we presented a new GRS approach that can be applied if no appropriate external weights are available and the marginal genetic effects are predominant, which means that the marginal genetic effects are stronger than the interaction effects [12]. In this approach, we used GRS with internal weights from the marginal genetic effects of the study itself and showed that using these GRS increased the power to detect gene-environment interactions substantially compared to the common single SNPs approach and to the usage of unweighted GRS with a well-controlled type I error [12]. In addition, GRS with weights from the marginal genetic effects estimated with elastic net regression [13] were able to handle a large number of correlated SNPs as well as noise SNPs, i.e. SNPs having no effect on the outcome of interest. Applying this approach to an epidemiological study, we showed in a study population of only 402 women that genetic variation in the endoplasmatic reticulum (ER) stress pathway might play a role in air pollution induced inflammation in the lung [7].

However, in scenarios with predominant interaction effects, a better approach might be to split the data into test and training data and using the training data to estimate the weights in the interaction term itself and the remaining test data to determine the GRS. Dudbridge (2013) evaluated a GRS approach in which the data were split into test and training data for the detection of marginal genetic effects [3]. Dudbridge recommended that the optimal balance of sample sizes between training and test data sets is close to one-half regardless of the proportion of noise SNPs or the *p*-value threshold [3]. Therefore, given an initial sample to be split into training and test subsets, an obvious rule of thumb is to make an even split [3]. However, to the best of our knowledge, this approach has never been evaluated for the detection of GxE interactions.

The aim of the current study is to present a new GRS approach for GxE interaction studies, called GRS-interaction-training, in which the weights are gained from the interaction terms in the training dataset that is split off the sample data and the remaining test data is used to determine the GRS. We performed a simulation study on the detection of gene-environment interactions in which we compared the performance of GRS-interaction-training to GRS with external weights (gold standard) and to weighted GRS-marginal-internal [12]. We considered scenarios with predominant marginal genetic effects and smaller additional GxE interaction effects, and vice versa. We simulated scenarios with an increasing number of noise SNPs (up to 200) and with varying minor allele frequencies.

Moreover, we applied these different weighting approaches to a real data set from the Traffic, Asthma and Genetics (TAG) Study (*N* = 4465 observations in a pooled dataset across six birth cohorts) concerned with investigating the role of genetic variation of the oxidative stress and inflammation pathway on air pollution-induced asthma at school age.

## Methods

### Determination of weighted GRS

Weighted GRS (*GRS*_*i*_) are defined as a weighted sums of the number of risk alleles (coded as 0, 1, 2) of *k* considered SNPs (*g*_*i*1_, …, *g*_*ik*_) for the *n* subjects (*i* = 1, …, *n*):

1 $$ {GRS}_i={w}_1\ {g}_{i1}+\dots +{w}_k\ {g}_{ik}. $$

The most common weighting approach is to use external weights *w*_1_, …, *w*_*k*_, e.g. marginal genetic effects of the *k* SNPs estimated in an independent study population [3, 11].

Genome-wide meta-analyses that provide the combined effect estimates of a range of independent studies are usually preferred, followed by meta-analyses, which only include a selected number of SNPs identified to be relevant for the phenotype and by GWAS in large single cohorts. Determining weights from two or more different external studies should be treated with caution because effect estimates from different cohorts are often incomparable, e.g. due to differences in study design, ethnicity or phenotype definitions.

A limitation of GRS with external weights is that we can only include SNPs for which the marginal genetic effects have been published. In this regard, GRS with external weights are usually restricted to SNPs with a genome-wide significant (p-value <5 × 10^−8^) marginal genetic effect in the external study population, whereas SNPs with a predominant interaction effect are usually not presented. Furthermore, not for every phenotype large-scale GWAS are published and sometimes they have been conducted only in populations with different ethnicity, sex or age range.

### GRS-marginal-internal approach

If no appropriate external weights are available, one approach that we developed recently is to estimate the weights *w*_1_, …, *w*_*k*_ from the internal marginal genetic effect of the study sample itself [12], called GRS-marginal-internal.

In this approach, the weights (${w}_1,\dots, {w}_k\Big)=\left({\widehat{\beta}}_1,\dots, {\widehat{\beta}}_k\right)$ in eq. (1) are estimated internally from a multivariate elastic net regression analysis [13–15] for the combined marginal genetic effect of *k* pathway-related SNPs on the health outcome *y* in the study population itself. In the elastic net regression model, the values of the unknown parameters for the intercept *β*_0_ and the marginal genetic effects of the *k* SNPs *β*_*j*_ (*j* = 1, …, *k*) can be estimated by minimizing the sum of the residual sum of squares and a penalty term:

2 $$ {\widehat{\beta}}_0,\widehat{\beta}={\displaystyle \begin{array}{c} argmin\\ {}{\beta}_0,\beta\ \end{array}}\left({\sum \limits}_{i=1}^n{\left({y}_i-{\beta}_0-{\sum \limits}_{j=1}^k{\beta}_j{G}_{ij}\right)}^2+P\left(\lambda, \beta \right)\right). $$

Here, *G* = (*g*_*i*1_, …, *g*_*ik*_) is an *n x k* matrix holding the *k* considered SNPs for the *n* subjects and the penalty function $P\left(\lambda, \beta \right):= \lambda {\sum}_{j=1}^k\left(\frac{1}{2}\left(1-\alpha \right)\kern0ex {\beta}_j^2+\alpha \kern0ex |{\beta}_j|\right)$ is a combined penalty of lasso and ridge regression penalties. We used cross-validation to find the optimal values of the regularization parameter *λ*, i.e. the largest *λ* –value such that the mean squared error (minMSE) is within 1 standard error (SE) of the minimum as implemented in the R package *glmnet* [14] and recommended in [15]. The penalty weight *α* can be chosen between 0 and 1. The elastic net with a penalty weight of *α* = 1 is identical to the lasso regression, whereas the elastic net with *α* = 0 is identical to the ridge regression [15]. Since we could show in our recent publication, that the penalty weight *α* only has a minor impact on power and type I error for the detection of interactions [12], we chose a penalty weight of *α* = 0.5 in this publication to receive a good balance between ridge and lasso regression. Zou and Hastie proposed the elastic net penalty for linear regression models [13] that was further extended to logistic regression and multinomial regression [14] and to the Cox regression [16].

### GRS-interaction-training approach

In scenarios with predominant interaction effects, i.e. in scenarios in which the GxE interaction effects are stronger than the marginal genetic effects, a better approach might be to use the coefficients from the interaction terms to determine the weights instead of using the marginal genetic effect estimates.

In this new approach, which we call GRS-interaction-training approach, SNPs get a larger weight to the extent that they interact more strongly with the environmental exposure.

Up to now, the use of training and test datasets for the construction of GRS has only been described for the detection of marginal genetic effects. If GRS are used to estimate marginal genetic effects, Dudbridge pointed out that the weights must be estimated from the marginal genetic effects in a training sample and be used to construct a GRS in an independent test dataset [3]. In the same line, Burgess et al. showed that using internal weights instead of weights from a training dataset should be avoided because it leads to biased effect estimates [17, 18].

Transferring this knowledge to GxE interaction analyses with GRS with weights from the interaction term itself, it is necessary to estimate these internal interaction weights in an independent training sample as well.

In the first step of the GRS-interaction-training approach, the initial sample is split randomly into a training dataset and a test dataset. Next, the elastic net regression is used to estimate the interaction parameters *δ*_*j*_ (*j* = 1, …, *k*) between each of the *k* SNPs and the environmental factor *E* by minimizing the sum of the residual sum of squares and a penalty term in the training data:

3 $$ {\widehat{\beta}}_0,\widehat{\beta},\widehat{\gamma},\widehat{\delta}\kern0.5em ={\displaystyle \begin{array}{c} argmin\\ {}{\beta}_0,\beta, \gamma, \delta\ \end{array}}\left({\sum}_{i=1}^n{\left({y}_i-{\beta}_0-{\sum}_{j=1}^k{\beta}_j{G}_{ij}-\gamma {E}_i-{\sum}_{j=1}^k{\updelta}_j{G}_{ij}{E}_i\right)}^2+P\left(\lambda, \beta, \gamma, \updelta \right)\right) $$

with *E* = (*e*_1_, …, *e*_*n*_) being an *n x* 1 matrix holding the considered environmental exposure *E* for the *n* subjects, the environmental effect parameter *γ* and the penalty function:

$$ P\left(\lambda, \beta, \gamma, \updelta \right):= \lambda \kern0ex \left({\sum \limits}_{j=1}^k\left(\left(\frac{1}{2}\left(1-\alpha \right)\kern0ex {\beta}_j^2+\alpha \kern0ex |{\beta}_j|\right)+\left(\frac{1}{2}\left(1-\alpha \right)\kern0ex {\updelta}_j^2+\alpha \kern0ex |{\updelta}_j|\right)\right)+\left(\frac{1}{2}\left(1-\alpha \right)\kern0ex {\gamma}^2+\alpha \kern0ex |\gamma |\right)\right). $$

The remaining parameters are defined as in eq. (2). The effect estimates for the interaction terms ${\widehat{\delta}}_j\ \left(j=1,\dots, k\right)$ are then used as weights *w*_1_, …, *w*_*k*_ for the GRS (see eq. (1) for the general definition of weighted GRS) in the remaining test data.

### Interaction analysis

In the subsequent gene-environment interaction analysis, a generalized linear model (GLM) [19, 20] is applied to estimate the gene-environment interaction (GRSxE interaction; interaction between GRS and environmental exposure) for the same health outcome *y* as in eqs. (2, 3). In a GLM, *y* is usually assumed to be generated from a distribution in the exponential family that includes, e.g., the normal, binomial, Poisson and gamma distribution. The mean *μ* of this distribution depends on the independent variables *X* through:

$$ E(Y)=\mu ={g}^{-1}\left( X\tau \right) $$

where *E*(*Y*) is the expected value of the random variable *Y*, *g* is the link function and *X* = (*grs*_*i*_,  *e*_*i*_,  *grs*_*i*_ *e*_*i*_) being an *n x* 3 matrix holding the considered GRS, the environmental exposure *E* and the interaction between the GRS and *E* for the *n* subjects. The unknown parameter vector *τ* is estimated using maximum likelihood.

## Simulation study

### Simulation design

The data for the simulation study was generated using the function simulateSNPglm from the R-package *scrime* [21]. Each of the simulated datasets contains six independent genetic risk factors (i.e. SNPs) and either 6, 50, 100, or 200 additional noise SNPs. The impact of more noise SNPs (up to 840) and highly correlated SNPs was discussed in our previous publication where we showed that weighted GRS with weights estimated in the elastic net regression can handle even a high number of noise and correlated SNPs very well [12]. In most scenarios, we randomly chose minor allele frequencies (MAF) between 0.01 and 0.45 for the six risk SNPs as well as for the noise SNPs. When analyzing the impact of the MAF, we varied the MAFs of the six risk SNPs between 0.01 and 0.45, whereas the MAFs for the noise SNPs were randomly selected. A dominant mode of inheritance was considered for each risk SNP.

We compared two scenarios:

In scenario (a), we constructed a predominant interaction effect which means that the interaction between each of the six risk SNPs and an environmental exposure *E* is set to an interaction effect of 1.5 with a smaller marginal genetic effect that is not explicitly defined (see [21]).

In scenario (b), we constructed a predominant marginal genetic effect, which means that the marginal genetic effect of each of the six risk SNPs is set to 1.5 with an additional (smaller) interaction effect. For the simulation of the gene-environment interaction terms in scenario (b), we followed the procedure previously described [12].

Effect estimates and *p*-values for the marginal genetic effects, the environmental effects and for the interaction effects of a simulated example dataset of *N* = 3000 are given for scenarios (a) and (b) in Tables S1 and S2 of Additional file 1.

### Simulation of external weights

In real data applications, it is often not or hardly possible to get appropriate external weights. Therefore, we simulated different types of external data with varying degrees of fit to the own study sample. First, external weights were estimated from the marginal genetic effects in an external dataset that was simulated from the same distribution as our study sample data (perfect weights). In addition, we simulated two scenarios with less appropriate external weights. In the first scenario, the effect estimates of the risk SNPs in our own study sample were larger than in the external data (underestimating weights) and in the second scenario, only one of the six risk SNPs of the external data was associated with the outcome in our own study sample (overestimating weights).

We simulated external data with the same sample size as in our own study sample and external data with a sample size being four times larger than in our own study sample and varied the number of noise SNPs from 6 to 200.

### Evaluation of power, proportion of sign-misspecification, and type I error

The main focus of the model comparison was to maximize the power to detect a gene-environment interaction with an acceptable type I error.

Power was evaluated in datasets with *N* = 3000 or *N* = 1000 observations and 100 or 1000 replications depending on the running time and precision needed in different scenarios. As shown in [12], the restriction to 100 replications only caused a minor sampling error of around 3%-points in power and type I error.

The power of the model was calculated as the proportion of times a true-positive interaction was correctly identified (sign of the parameter estimate for the GRSxE interaction term correctly identified and p-value < 0.05) across all replications. The type I error of the model was calculated as the proportion of times a false-positive interaction was identified under the null hypothesis. We further evaluated the proportion of sign-misspecifications, which was calculated as the proportion of times a significant interaction was identified, but the sign of the parameter estimate for the GRSxE interaction term was not correctly determined.

Within the evaluation of our GRS-interaction-training approach, we investigated the optimal balance between training and test datasets by comparing different proportions: We started with the scenario recommended by Dudbridge (2013) for GRS used for the detection of marginal genetic effects [3], in which the training and the test datasets have an even sample size (1:1). Further scenarios are based on smaller training datasets (1:2, 1:3, 1:4, 1:9 and 1:19) and larger training datasets (19:1, 9:1, 4:1, 3:1, 2:1) than test datasets.

All analyses were performed using R 3.3.1 [22].

## Results

### Simulation study

#### GRS-interaction-training approach – Balance between training vs. test data

In a first step, we evaluated the optimal balance between training and test data applying our GRS-interaction-training approach.

**Fig. 1:**
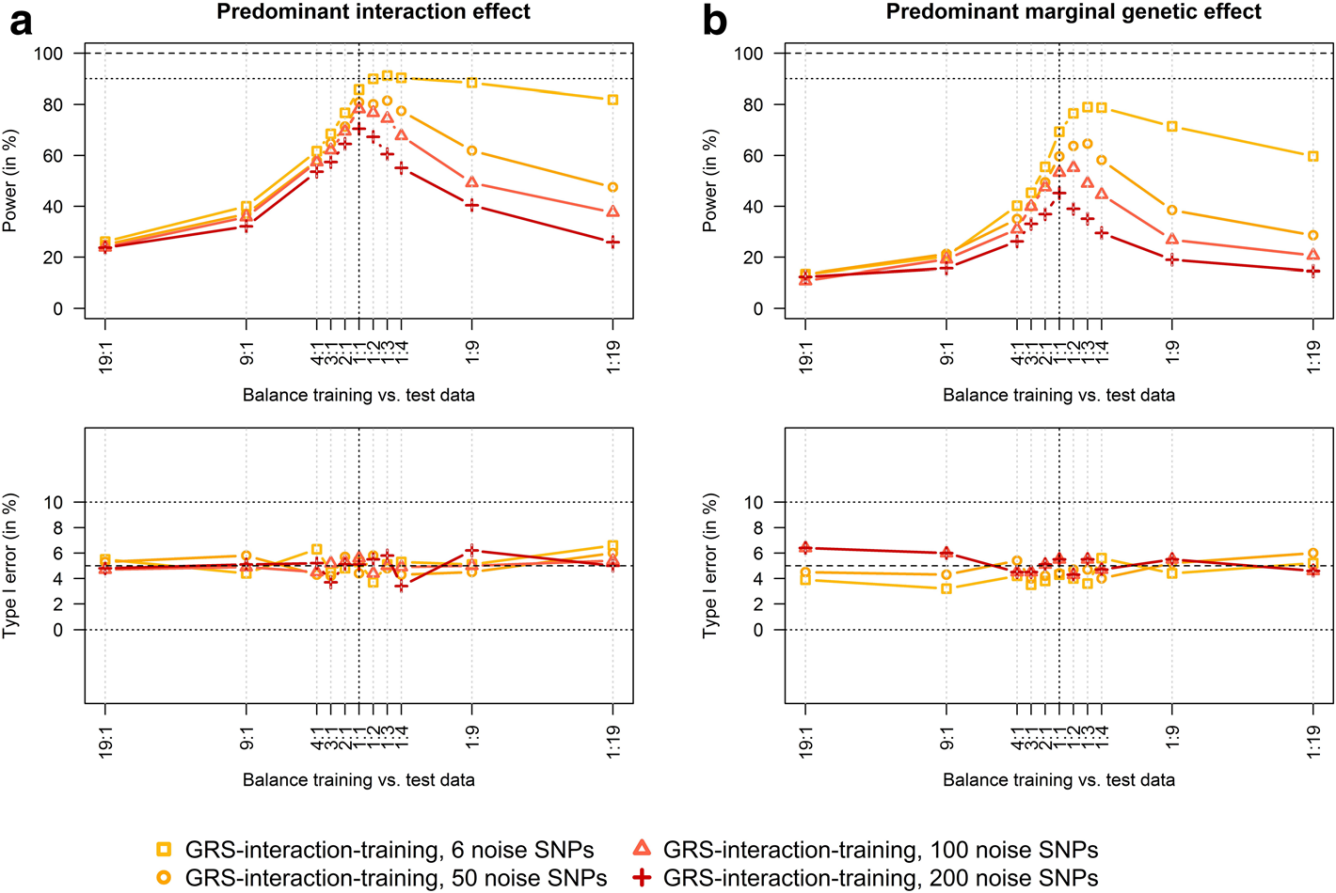
Impact of the balance between training vs. test data on power and type I error of the GRS-interaction-training approach. Scenarios with predominant interaction effects (**a**) and predominant marginal genetic effects (**b**). Balance training vs. test data increases from 19:1 to 1:19, scenarios with 6 risk SNPs that interact with the environmental exposure and 6, 50, 100 and 200 additional noise SNPs that are not associated with the outcome (*N* = 3000 observations and 1000 replications)

In Fig. 1, power and type I error to detect GxE interactions for (a) predominant interaction effects and (b) predominant marginal genetic effects are presented. Power and type I error were evaluated with an increasing sample size of the training data in comparison to the test data (from 19:1 to 1:19).

This figure reveals that in scenarios with many noise SNPs, the optimal split is close to one-half and the balance is roughly symmetrical around one-half. However, with a decreasing number of noise SNPs, a higher power was achieved by increasing the test data in comparison to the training data. In scenarios with an equal number of noise and risk SNPs, i.e. with six noise and six risk SNPs, the optimal balance between training and test data lay between 1:3 and 1:4. The type I error was well controlled over all scenarios and there was no difference in power and type I error between scenarios with predominant interaction effects (Fig. 1a) and scenarios with predominant marginal genetic effects (Fig. 1b).

### GRS-interaction-training in comparison to previous weighting approaches

Next, we compared the GRS-interaction-training approach (balance training vs. test data 1:1) to our previously published GRS-marginal-internal approach [12] and to GRS with external weights (which is typically considered as gold standard) in scenarios with (a) predominant interaction effects and (b) predominant marginal genetic effects with an increasing number of noise SNPs (up to 200).

**Fig. 2:**
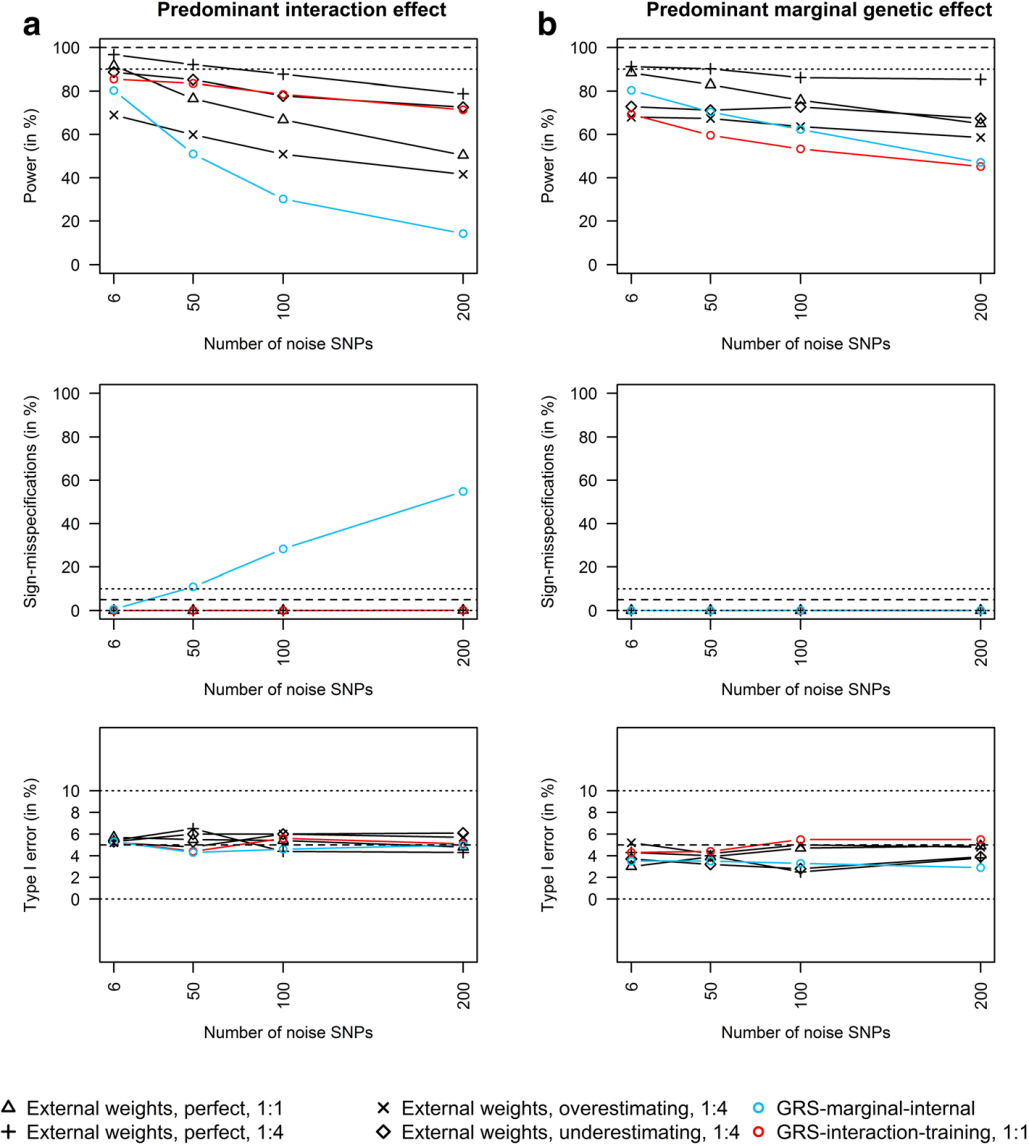
External vs. internal weights with increasing number of noise SNPs (up to 200) in scenarios with predominant interaction effects (a) and predominant marginal genetic effects (b). Power, sign-misspecifications and type I error comparison of i) the GRS-interaction-training approach (red lines; one half of the data used as training data and the other half as test data), ii) the GRS-marginal-internal approach (blue lines) and iii) GRS with external weights (black lines). We compared three types of external weights. Perfect: data from the same distribution as the sample data; overestimating: only one of the six risk SNPs of the external data was associated with the outcome in the sample data; underestimating: effect estimates of the risk SNPs in the sample data were 30% larger than in the external data). External weights with “1:1” and “1:4”: Balance between size of sample data vs. size of external data (*N* = 3000 observations and 1000 replications)

In scenarios with predominant interaction effects (see Fig. 2a), the GRS-interaction-training approach achieved a higher power than the GRS-marginal-internal approach. In particular, in scenarios with many noise SNPs, the GRS-marginal-internal approach reached a very low power to detect interaction effects. Furthermore, with more noise, there was a high number of sign-misspecifications when using the GRS-marginal-internal approach in scenarios with predominant interaction effects.

In scenarios with predominant marginal genetic effects (see Fig. 2b), the GRS-marginal-internal approach achieved a slightly higher power to detect interaction effects than the GRS-interaction-training approach, but the differences became smaller with an increasing number of noise SNPs. There were no sign-misspecifications in scenarios with predominant marginal genetic effects.

GRS with perfect external weights that were gained from external data that were simulated from the same distribution as our study sample data, outperformed the GRS-interaction-training and the GRS-marginal-internal approaches. However, if the sample size of the external data was not larger than our own study sample size, the GRS-interaction-training approach achieved a higher power than GRS with perfect external weights in scenarios with predominant interaction effects (Fig. 2a).

Furthermore, in real data applications, there is usually no perfect match between the external data and the sample data, e.g., effect estimates in the own study sample might differ from those in the external data or only a subset of risk SNPs identified in the external data is associated with the outcome in the own study sample. In these scenarios, the GRS-interaction-training approach was often more appropriate to detect predominant interaction effects than GRS with external weights. The GRS-marginal-internal approach only outperformed GRS with external weights in the detection of predominant marginal genetic effects if there were <100 noise SNPs in the data (Fig. 2b).

The type I error was well controlled over all scenarios (Fig. 2).

### GRS-interaction-training vs. GRS-marginal-internal – Impact of MAF

In a last step, we analyzed the impact of the MAFs of the six risk SNPs on power, proportion of sign-misspecifications and type I error of the GRS-interaction-training approach in comparison to the GRS-marginal-internal approach.

**Fig. 3:**
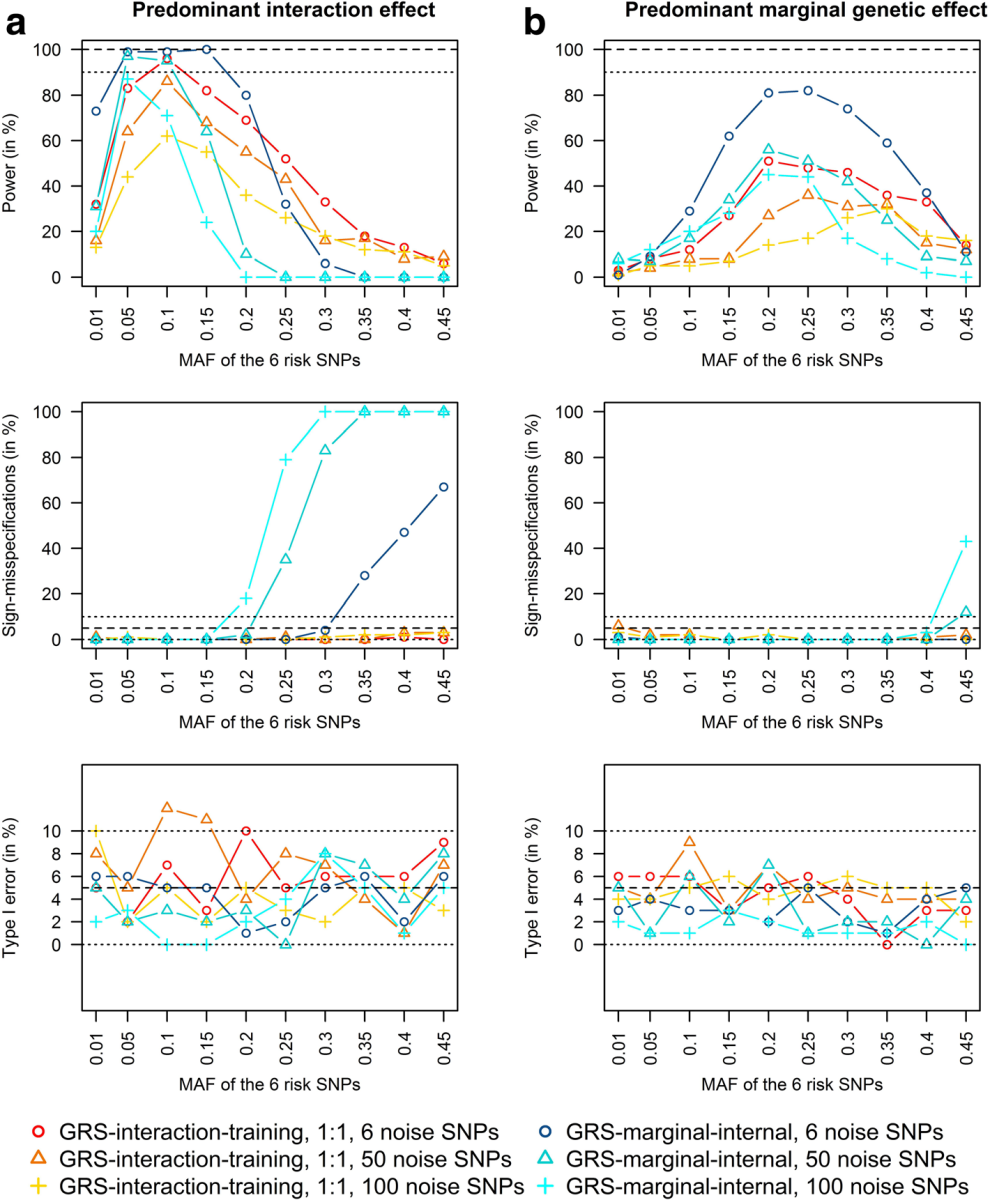
Power, sign-misspecifications and type I error comparison of the GRS-interaction-training approach (one half of the data used as training data and the other half as test data) vs. the GRS-marginal-internal approach. Scenarios with predominant interaction effects (a) and predominant marginal genetic effects (b). Minor allele frequencies of the 6 risk SNPs increase from 0.01 to 0.45, scenarios with 6, 50 and 100 noise SNPs (*N* = 1000 observations and 100 replications)

In scenarios with a predominant interaction effect (see Fig. 3a), the power achieved by the GRS-interaction-training approach was highest for MAFs between 0.05 and 0.20. Furthermore, there were no sign-misspecifications and the type I error was well controlled. The power achieved by the GRS-marginal-internal approach was even higher than the power achieved by the GRS-interaction-training approach in scenarios with only a small number of noise SNPs and small MAFs. However, with more noise and MAFs >0.1, the GRS-interaction-training approach outperformed the GRS-marginal-internal approach. Most interestingly, there was a high number of sign-misspecifications in scenarios with MAFs ≥0.2 when applying the GRS-marginal-internal approach, especially in scenarios with many noise SNPs.

In scenarios with a predominant marginal genetic effect (see Fig. 3b), the GRS-marginal-internal approach achieved a higher power than the GRS-interaction-training approach with an acceptable proportion of sign-misspecifications.

The type I error was well controlled in all scenarios, but with a higher variation due to the reduced number of replications (100 instead of 1000).

### Real data application

The real data application was based on a dataset from the Traffic, Asthma and Genetics (TAG) Study (*N* = 4465 observations in the pooled dataset across six birth cohorts) in which the interaction between air pollution and SNPs associated with oxidative stress and inflammation on incident childhood asthma was investigated.

Traffic-related air pollution, asthma, SNPs, and potential confounder data were pooled across six birth cohorts. Parents reported physician-diagnosed asthma from birth to 7–8 years of age (confirmed by pediatric allergist in two cohorts). Individual estimates of annual average air pollution [nitrogen dioxide (NO_2_), particulate matter ≤2.5 μm (PM_2.5_), PM_2.5_ absorbance, ozone] were assigned to each child’s birth address using land use regression, atmospheric modeling, and ambient monitoring data. Gene-environment interactions between air pollution and SNPs in *GSTP1* (rs1138272 and rs1695) and *TNF* (rs1800629) on asthma were investigated.

The main findings of the pooled analyses were that NO_2_ (OR = 1.23; 95%-CI: 1.03, 1.46, for a 10-μg/m^3^ increase in NO_2_) and *GSTP1* rs1138272 (TT/TC vs. CC; OR = 1.49; 95%-CI: 1.20, 1.84) were marginally associated with asthma and a significant interaction between *GSTP1* rs1138272 and NO_2_ on asthma was detected (Bonferroni-corrected *p* = 0.012) [23].

More information about the TAG study can be found in [23–25].

In our analysis, we focused on the German Infant Study on the influence of Nutritional Intervention plus environmental and genetic influences of on allergy development (GINIplus) as study sample (*N* = 593 observations), which is one of the six birth cohorts included in the TAG study. We compared the *p*-values derived from weighted GRS with weights from the pooled analysis as published in [23] (proxy for external weights) to p-values from the GRS-marginal-internal approach and to p-values from the GRS-interaction-training approach (balance training vs. test data 1:1 (*N*_test_ = 296), 1:2 (*N*_test_ = 395) and 1:3 (*N*_test_ = 444)).

In Table 1, an overview on the marginal genetic effects in the pooled analysis [23] and in GINIplus are given. Only the marginal genetic association between *GSTP1* rs1138272 and asthma was significant in the pooled TAG analysis. Effect estimates differed only slightly between the pooled analysis and GINIplus, being ~30% stronger in GINIplus than in the pooled analysis. However, due to the small sample size of GINIplus (*N* = 593), this marginal association was not significant in GINIplus.

**Table 1:** Real data application. Marginal genetic effects for the associations of three *GSTP1* & *TNF* SNPs with parents reported physician-diagnosed asthma from birth to 7–8 years of age in the pooled TAG data and in GINIplus considering a dominant mode of inheritance for the three SNPs

		Association with asthma		
		*N*	OR^a^	*p*-value^b^
*GSTP1* rs1138272	Pooled^c^	4465	1.49	<0.001
	GINIplus^d^	593	1.67	0.348
*GSTP1* rs1695	Pooled^c^	4635	0.91	0.430
	GINIplus^d^	593	0.75	0.972
*TNF* rs1800629	Pooled^c^	4356	1.04	0.647
	GINIplus^d^	593	0.80	1.000

^a^Adjusted for study, city, intervention, infant sex, maternal age at birth, maternal smoking during pregnancy, environmental tobacco smoke in the home, birth weight, and parental atopy. ^b^p-values were corrected for multiple testing using the Bonferroni method (raw *p*-values multiplied by the number of analyzed SNPs (3)). ^c^Pooled data from BAMSE, CAPPS, GINIplus, LISAplus, SAGE and PIAMA, *N*, ORs and p-values as published in MacIntyre et al. (2014). ^d^determined for this publication

Table 2 shows the results of the GxE interaction analysis in GINIplus. The significant GxE interaction between *GSTP1* rs1138272 and NO_2_ on asthma, which was identified in the pooled analysis [23], was identified by each GRS approach. The lowest *p*-values were achieved by applying the GRS-marginal-internal approach and GRS with external weights, followed by the GRS-interaction-training (using 25% of the data for training and the remaining 75% as test data). The weights from the GRS-marginal-internal approach were almost identical to the univariate estimates from the pooled analysis. The GRS-interaction-training approach was the only approach that correctly identified *GSTP1* rs1138272 as the only SNP that interacts with air pollution (cf. [23]) by setting the weights of the other SNPs to zero.

**Table 2:** Real data application. GxE interaction analysis in GINIplus between a GRS of three *GSTP1* & *TNF* SNPs and air pollution exposure (NO_2_) with parents reported physician-diagnosed asthma from birth to 7–8 years of age

		Weights for GRS			GRSxE interaction	
	*N*	*GSTP1* rs1138272	*GSTP1* rs1695	*TNF* rs1800629	OR^a^	*p*-value
GRS with weights from pooled marginal genetic effects^b^	593	ln(1.49) ≈ 0.40	ln(0.91) ≈ −0.09	ln(1.04) ≈ 0.04	16.31	0.004
GRS-marginal-internal^c^	593	0.69	−0.09	0.00	8.83	0.004
GRS-interaction-training (1:1)^d,e^	296	0.63	0.00	0.00	9.71	0.028
GRS-interaction-training (1:2)^d,f^	395	0.64	0.00	0.00	9.24	0.014
GRS-interaction-training (1:3)^d,g^	444	0.85	0.00	0.00	7.34	0.007

^a^OR and p-values for the interaction effects. Adjusted for study, city, intervention, infant sex, maternal age at birth, maternal smoking during pregnancy, environmental tobacco smoke in the home, and parental atopy. ^b^Pooled data from BAMSE, CAPPS, GINIplus, LISAplus, SAGE and PIAMA; ln(ORs) as published in MacIntyre et al. (2014) were used as weights (compare Table 1). ^c^estimated in GINIplus within this publication, estimates from the elastic net regression (α = 0.5) for the marginal genetic effects in GINIplus. ^d^Weights from the interaction term itself when using parts of the data to estimate the weights and the remaining data to determine the GRS. ^e^Balance training vs. test data 1:1. ^f^Balance training vs. test data 1:2. ^g^Balance training vs. test data 1:3

## Discussion

In this article, we presented a new weighting approach, called GRS-interaction-training, for GRSxE interaction studies in which parts of the study sample are used to estimate the weights and the remaining data are employed to determine the GRS.

In a simulation study and a subsequent real data application, we compared the performance of this approach to weighted GRS with internal weights from the marginal genetic effects, called GRS-marginal-internal [12], and GRS with external weights for the detection of gene-environment interactions.

Our simulation study has shown that the power for detecting GxE interactions reached by applying the GRS-interaction-training approach was only slightly lower than the power achieved by weighted GRS with external weights from the marginal genetic effects estimated in an independent study population that fits perfectly to our own study sample. If the external data, however, did not fit to the own study sample perfectly or the sample size of the external data was not larger than our own sample size, the power was higher when using the GRS-interaction-training approach.

The sample size of the test data in the GRS-interaction-training approach is only half of the sample size from the GRS-marginal-internal approach, because in the GRS-interaction-training approach half of the data is used to determine the weights and the remaining test data to calculate the GRS and to estimate the interaction. Nevertheless, if there were no external weights available and the underlying GxE interaction effect was larger than the marginal genetic effect, the highest power was reached with the GRS-interaction-training approach. If the underlying marginal genetic effect was substantially larger than the GxE interaction effect, the GRS-marginal-internal approach was more appropriate.

### GRS-interaction-training approach – Balance between training vs. test data

Motivated by the idea that the interaction itself might be more suitable to estimate the weights than the marginal genetic effect, we divided each of our datasets into a training and a test dataset and used the interaction estimates from the training data as weights for the GRS in the test data. Dudbridge (2013) evaluated a similar approach for the detection of marginal genetic effects and reported that the optimal balance of sample sizes between training and test datasets is close to one-half regardless of the proportion of noise SNPs or the p-value threshold [3]. In our study, this recommendation showed up to be true for scenarios with many noise SNPs (e.g., 6 risk SNPs and 200 noise SNPs) and the balance was roughly symmetrical around one-half which is also in line with [3]. However, in contrast to Dudbridge (2013), with a decreasing number of noise SNPs (down to only 6), a higher power was achieved by increasing the size of the test data proportionally to the size of the training data. This finding was confirmed in our real data application with only two noise SNPs and one risk SNP, as a lower p-value was achieved when using more test data than training data. Nevertheless, since we usually consider a large number of noise SNPs in most gene-environment interaction studies, we generally support Dudbridge’s rule of thumb to make an even split between training and test data for GxE interaction studies.

### Internal vs. external weights

Our simulation study has confirmed that the gold standard for the construction of GRS is to use external weights, e.g., from the marginal genetic effects estimated in independent study populations, if the external data fit very well to the study sample. This strong assumption means that the marginal genetic associations in the external data are the same as in our own study sample, this might but must not be reached if the phenotype is assessed in exactly the same way and that there is no ethnic or age difference between the study populations. In real data analyses, these assumptions are often not fulfilled because large scale GWAS are not published for every phenotype and sometimes only in populations with different ethnicity, sex or age range.

The violation of these assumptions might lead to a decrease of power for detecting interaction effects with GRS with external weights. Therefore, in the practical analysis of real data, using internal weights from the study population itself might often be a more powerful alternative to detect GxE interactions.

However, in our real data application, the power reached by GRS with external weights was similar to the power reached by the two approaches with internal weights. One reason for that might be that our study sample (GINIplus) was included in the estimation of the “external” effects. Therefore, the effect estimates from the pooled analysis might fit slightly better to the GINIplus data than they would have fitted if the GINIplus data would not have been part of the pooled analysis. Furthermore, a limitation of the GRS-interaction-training approach is that the GRSxE interaction term can only be estimated in a subset (i.e. the test data) of the original sample data which reduces the power to detect interactions.

A major limitation of GRS with external weights is that we can only include SNPs for which the marginal genetic effects have been published. In this regard, GRS with external weights are usually restricted to SNPs with a genome-wide significant (p-value <5 × 10^−8^) marginal genetic effect in the external study population, whereas SNPs with a predominant interaction effect are usually not presented. For GxE interaction studies, this leads to a publication bias towards SNPs with predominant marginal genetic effects. To avoid this publication bias and to increase the power for detecting GxE interactions, estimates from genome wide gene-environment interaction studies might be used. However, up to now, very few genome-wide gene-environment interaction studies have been published because of the limited power to detect interactions in genome-wide analyses.

From a biological perspective, a pathway-orientated GxE interaction analysis might be a more powerful and biologically plausible alternative to genome-wide approaches. Very recently, we could, e.g., show in a study population consisting of 402 women that genetic variations in the ER stress pathway might play a role in air pollution induced inflammation in the lung using the GRS approach with internal weights from the marginal genetic effects, although there was no significant marginal genetic effect on the individual SNP level [7].

### GRS-interaction-training vs. GRS-marginal-internal

In scenarios with a predominant interaction effect, i.e. an interaction effect that is (substantially) larger than the marginal genetic effect, the GRS-interaction-training approach was more powerful than the GRS-marginal-internal approach, particularly in the presence of noise SNPs. Furthermore, applying the GRS-marginal-internal approach in scenarios with predominant interaction effects might lead to a high number of sign-misspecifications when the MAFs of the risk SNPs are ≥0.2 and in the presence of noise.

However, in scenarios with a predominant marginal genetic effect and a smaller additional interaction effect, the GRS-marginal-internal approach achieved a slightly higher power than GRS-interaction-training approach with an acceptable number of sign-misspecifications.

In real data applications, the decision if the interaction or the marginal genetic effect is predominant, should be made a priori and be based on biological knowledge. If the SNPs were chosen because the underlying genes had been identified to be marginally associated with the same or a related phenotype (e.g. in a large-scale genome-wide meta-analysis), independently of the environmental exposure, the weights should be determined from the marginal genetic effects (GRS-marginal-internal). Nevertheless, if the SNPs were chosen because of their potential impact on the biological mechanisms mediating the association between the environmental exposure and disease development, the weights should be determined from the interaction term (GRS-interaction-training approach). Either this knowledge might be based on mechanistic studies or on epigenome-wide association studies (EWAS). EWAS present differentially methylated probes (DMPs) and regions (DMRs) in balance to disease outcomes (e.g. [26] for lung function). Since EWAS identify regions that are modified by environmental factors, they might provide a good pre-selection of genetic regions to be considered in GxE interaction studies.

In the TAG study, e.g., the considered SNPs were chosen, as the biological mechanisms were thought to underlie both the toxicity of traffic-related air pollution and the development of asthma [27]. This was confirmed by our performed analysis, which shows that the GRS-marginal-internal approach reached almost the same power as GRS-interaction-training approach.

### Strengths and limitations

Our study has several strengths. To our knowledge this is the first study presenting GRS with weights from the interaction term itself and comparing GRS with internal vs. external weights for the detection of gene-environment interactions. Furthermore, this is the first study comparing interaction approaches in scenarios with predominant interaction vs. predominant marginal genetic effects, a differentiation that is often ignored in the real data practice but which was shown to have a major impact on the selection of the most powerful analytic strategy. A further strength is that we analyzed the performance of the GRS approaches in the presence of noise and SNPs with different MAFs to cover several data structures common in GxE interaction studies.

A few limitations and outstanding issues should be noted. In our simulation study, we compared the performance of GRS with internal and external weights in quite simple scenarios, which might not cover all types of interaction models. We did not include different modes of inheritance, gene-gene or other more complex interactions in these scenarios. Such considerations might be beneficial to further optimize the weighted GRS for other scenarios.

Moreover, a comparison of the considered GRS approaches with other state-of-the-art interaction approaches might be interesting. However, as Aschard recently showed, the use of GRS can increase the power to detect GxE interactions in comparison to common univariate single-variant approaches and the joint test of main genetic and interaction effects [4, 5]. We additionally compared our GRS approaches with a multiple logistic lasso regression considering *p*-values estimated using the significance test for the lasso [28]. The results of this comparison presented in Additional file 1 show that our GRS approaches outperform the results of a lasso regression in the considered scenarios.

Furthermore, there is room for improvement regarding the decision making process between a predominant interaction effect and a predominant marginal genetic effect because detailed a priori knowledge about the biological pathways is often limited. One possibility to improve the a-priori knowledge might be to use information from EWAS. The growing field of epigenetics might clarify many of the biological pathways how environmental exposures might induce health problems and thereby improve the selection process of candidate SNPs for pathway based GxE interaction studies. A possibility to improve the GRS approaches might be to combine the GRS-marginal-internal approach and the GRS-interaction-training approach to reach a good power for the detection of interactions in scenarios with predominant marginal genetic effects as well as in scenarios with predominant interaction effects.

Our real data application has the limitation that we could only include the three SNPs from which we had previous knowledge about the marginal genetic and interaction effects in a large pooled analysis [23]. However, this is often a limitation in the daily practice as well, since external weights are often limited to, e.g., genome-wide significant SNPs because other effect estimates are often not reported. Furthermore, since GINIplus (*N* = 593) was part of the TAG consortia (*N* = 4465), the weights from the pooled marginal genetic effects were not independent from our sample data. However, this problem does also often occur in the real data practice because large scale genome-wide meta-analyses often include all study populations that are available for the considered phenotype and thereby often include the own study sample as well.

## Conclusion

In conclusion, when no appropriate external weights are available (due to, e.g., ethnic differences or differences in the phenotype assessment), we recommend to use internal weights from the study population itself to construct weighted GRS for GxE interaction studies. If the SNPs were chosen because a marginal genetic effect was hypothesized, the weights should be estimated from the marginal genetic effects (GRS-marginal-internal approach). If the SNPs were chosen because of their potential impact on the biological mechanisms mediating the association between the environmental exposure and disease development, the weights should be estimated from the interaction term itself in a training dataset (GRS-interaction-training approach).

## Additional file

Additional file 1: Table S1. Simulated predominant interaction effects (example data for *N* = 3000). **Table S2.** Simulated marginal genetic effects (example data for N = 3000). Comparison of GRS approaches and lasso regression. **Figure S1.** Power and sign-misspecifications comparison. **Figure S2.** Type I error comparison. (DOCX 405 kb)

## Abbreviations

EWAS: Epigenome-wide association study
GINIplus: German Infant Study on the influence of Nutritional Intervention plus environmental and genetic influences of on allergy development
GLM: Generalized linear model
GRS: Genetic risk score
GRSxE interaction: Interaction between GRS and environmental exposure
GWAS: Genome-wide association study
GxE interaction: Gene-environment interaction
MAF: Minor allele frequency
NO_2_: Nitrogen dioxide
PM_2.5_: Particulate matter ≤2.5 μm
SNP: Single nucleotide polymorphism
TAG Study: Traffic, Asthma and Genetics Study
