# Supplementary material for: Comparison of weighting approaches for genetic risk scores in gene-environment interaction studies
Source: BMC Genet. 2017 Dec 16;18:115. doi: 10.1186/s12863-017-0586-3 (PMC5732390; doi:10.1186/s12863-017-0586-3)
Supplement: Additional file 1: Table S1. — Simulated predominant interaction effects (example data for N = 3000). Table S2. Simulated marginal genetic effects (example data for N = 3000). Comparison of GRS approaches and lasso regression. Figure S1. Power and sign-misspecifications comparison. Figure S2. Type I error comparison. (DOCX 405 kb) [file 12863_2017_586_MOESM1_ESM.docx]

**Additional file 1**

**Comparison of weighting approaches for genetic risk scores in gene-environment interaction studies**

**Authors**

Anke Hüls^1,2^, Ursula Krämer^1^, Christopher Carlsten^3,4,5^, Tamara Schikowski^1^, Katja Ickstadt^2^*, Holger Schwender^6^*

1: IUF-Leibniz Research Institute for Environmental Medicine, Düsseldorf, Germany.

2: Faculty of Statistics, TU Dortmund University, Dortmund, Germany

3: Department of Medicine, University of British Columbia, Vancouver, British Columbia, Canada

4: Institute for Heart and Lung Health, Vancouver, British Columbia, Canada

5: School of Population and Public Health, University of British Columbia, British Columbia,

Canada.

6: Mathematical Institute, Heinrich Heine University, Düsseldorf, Germany

* equal contribution

**Corresponding author**

Anke Hüls, IUF-Leibniz Research Institute for Environmental Medicine, Auf’m Hennekamp 50, 40225 Düsseldorf, Germany, Phone number: +49 211 3389 293, E-Mail: Anke.Huels@IUF-Duesseldorf.de

| **Table S1:** Simulated Predominant interaction effects (example data for N=3,000). Effect estimates (odds ratios; OR) and (raw) p-values for the marginal genetic effects (G), the environmental effects (E) and the interaction terms (GxE). Results from a single SNPs analysis. | | | | | | | | |
| --- | --- | --- | --- | --- | --- | --- | --- | --- |
|  | **SNP** | **MAF** | **OR (G)** | **p-value (G)** | **OR (E)** | **p-value (E)** | **OR (GxE)** | **p-value (GxE)** |
| **6 risk SNPs** | SNP1 | 0.35 | 1.08 | 0.234 | 17.79 | <0.001 | 3.56 | <0.001 |
|  | SNP2 | 0.42 | 1.07 | 0.293 | 17.79 | <0.001 | 2.9 | <0.001 |
|  | SNP3 | 0.37 | 1.12 | 0.076 | 17.79 | <0.001 | 4.51 | <0.001 |
|  | SNP4 | 0.44 | 1.28 | <0.001 | 17.79 | <0.001 | 2.62 | <0.001 |
|  | SNP5 | 0.23 | 1.15 | 0.062 | 17.79 | <0.001 | 5.45 | <0.001 |
|  | SNP6 | 0.09 | 0.92 | 0.398 | 17.79 | <0.001 | 2.8 | 0.004 |
| **6 noise SNPs** | SNP7 | 0.16 | 1.08 | 0.348 | 17.79 | <0.001 | 1.59 | 0.054 |
|  | SNP8 | 0.26 | 0.97 | 0.656 | 17.79 | <0.001 | 0.93 | 0.687 |
|  | SNP9 | 0.37 | 0.92 | 0.198 | 17.79 | <0.001 | 0.8 | 0.165 |
|  | SNP10 | 0.48 | 1.07 | 0.271 | 17.79 | <0.001 | 1.18 | 0.310 |
|  | SNP11 | 0.03 | 1.01 | 0.971 | 17.79 | <0.001 | 0.88 | 0.766 |
|  | SNP12 | 0.09 | 1.21 | 0.087 | 17.79 | <0.001 | 1.42 | 0.279 |

| **Table S2:** Simulated Predominant marginal genetic effects (example data for N=3,000). Effect estimates (odds ratios; OR) and (raw) p-values for the marginal genetic effects (G), the environmental effects (E) and the interaction terms (GxE). Results from a single SNPs analysis. | | | | | | | | |
| --- | --- | --- | --- | --- | --- | --- | --- | --- |
|  | **SNP** | **MAF** | **OR (G)** | **p-value (G)** | **OR (E)** | **p-value (E)** | **OR (GxE)** | **p-value (GxE)** |
| **6 risk SNPs** | SNP1 | 0.36 | 1.54 | <0.001 | 1.09 | <0.001 | 1.01 | 0.047 |
|  | SNP2 | 0.43 | 1.45 | <0.001 | 1.09 | <0.001 | 1.02 | 0.001 |
|  | SNP3 | 0.37 | 1.47 | <0.001 | 1.09 | <0.001 | 1.02 | 0.004 |
|  | SNP4 | 0.43 | 1.66 | <0.001 | 1.09 | <0.001 | 1.01 | 0.101 |
|  | SNP5 | 0.24 | 1.24 | <0.001 | 1.09 | <0.001 | 1.03 | <0.001 |
|  | SNP6 | 0.09 | 1.08 | 0.428 | 1.09 | <0.001 | 1.03 | 0.004 |
| **6 noise SNPs** | SNP7 | 0.16 | 0.96 | 0.592 | 1.09 | <0.001 | 1.00 | 0.546 |
|  | SNP8 | 0.25 | 1.13 | 0.036 | 1.09 | <0.001 | 0.99 | 0.200 |
|  | SNP9 | 0.37 | 1.03 | 0.632 | 1.09 | <0.001 | 1.00 | 0.851 |
|  | SNP10 | 0.50 | 1.11 | 0.050 | 1.09 | <0.001 | 1.01 | 0.013 |
|  | SNP11 | 0.02 | 1.11 | 0.538 | 1.09 | <0.001 | 1.00 | 0.846 |
|  | SNP12 | 0.09 | 1.01 | 0.930 | 1.09 | <0.001 | 0.99 | 0.492 |

**Comparison of GRS approaches and lasso regression**

**Methods**

The GRS approaches are further compared to a lasso logistic regression with interaction terms (equation (3) in the main text with $\alpha=1$) applied to the whole study sample. Lockhart et al. recently proposed a test statistic based on lasso fitted values, called the covariance test statistic [1]. The covariance test statistic is constructed from the lasso solution path that can be computed by the LARS algorithm of Efron et al. [2]. The significance test for the lasso is implemented in the R package *covTest* [1]. Using this R package, p-values can be generated for the coefficients $\hat{\delta}_{j} (j=1, \ldots, k)$ of the interaction terms in equation (3) in the main text.

The power of the model was calculated as average proportion of true-positive interactions that were correctly identified within the six risk SNPs (sign of the parameter estimate for the interaction term correctly identified and p-value<0.05) across 100 replications. The type I error of the model was calculated as the number of false-positive interactions that were identified within the noise SNPs in the scenarios with six risk SNPs that interact with the environmental factor $E$ as well as under the null hypothesis (no interaction present) across 100 replications. We further evaluated the proportion of sign-misspecifications, which was calculated as the proportion of times a significant interaction was identified, but the sign of the parameter estimate for the interaction term was not correctly determined.

**Results**

We compared the GRS-interaction-training approach (balance training vs. test data 1:1) and the GRS-marginal-internal approach [3] to the results of the lasso regression in scenarios with (A) predominant interaction effects and (B) predominant marginal genetic effects with an increasing number of noise SNPs (up to 100). Over all scenarios, the lasso regression reached a lower power to detect interactions than the GRS-approaches (Figure S1, Additional file 1). Furthermore, the capacity of the lasso regression to differentiate between true and false positive SNPs was low in scenarios with a high number of noise SNPs (e.g. 100 noise SNPs and six risk SNPs; compare type I error in Figure S2, Additional file 1). Furthermore, in the presence of six risk SNPs with predominant interaction effects and a high number of noise SNPs, the number of false positives SNPs showed a large variation across the 100 replications, e.g. ranging from 0 to 53 in the presence of 100 noise SNPs. This variation is presented in the boxplots in Figure S2.


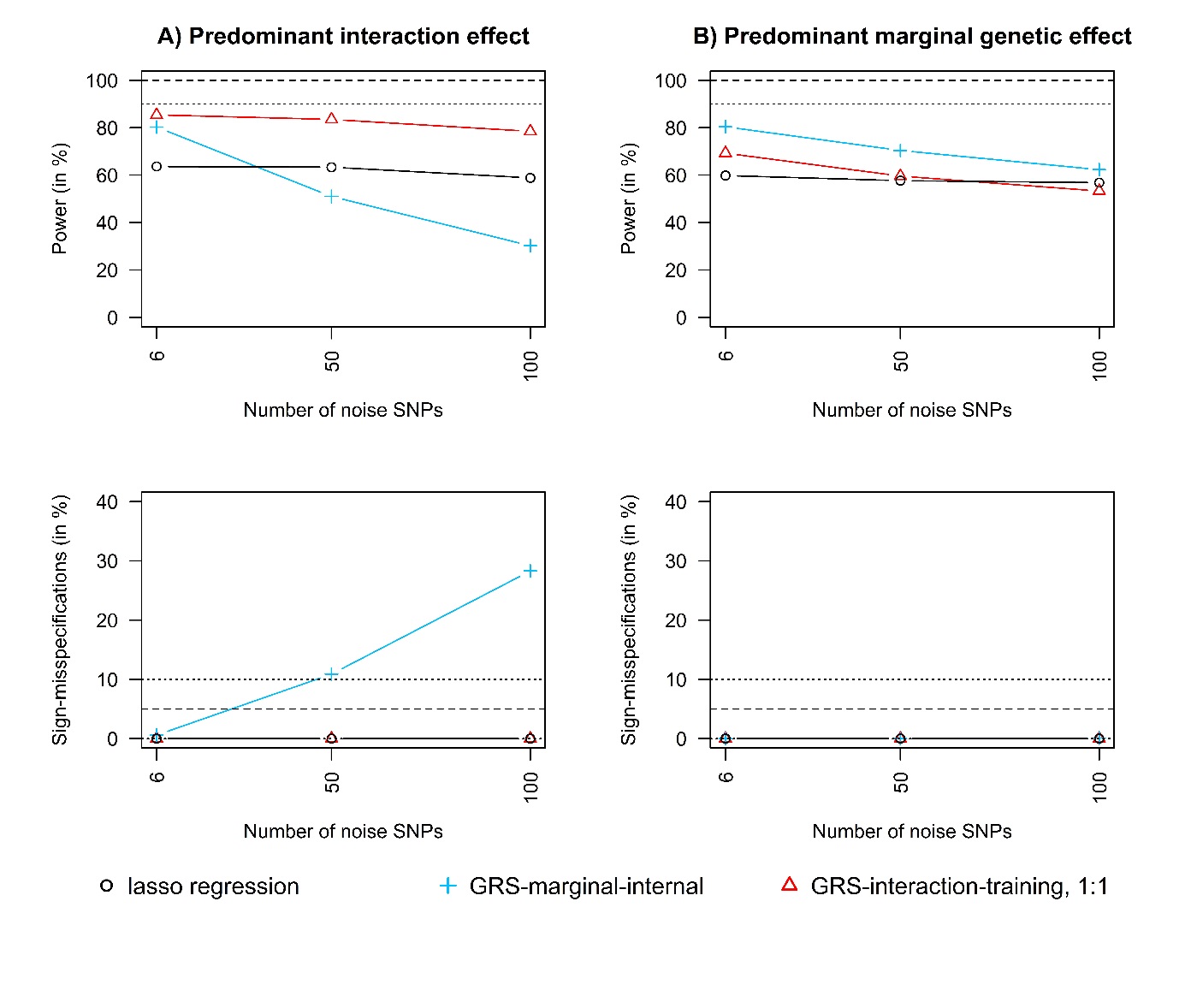


**Figure S1:** GRS-approaches vs. lasso regression with increasing number of noise SNPs (up to 100). Power and sign-misspecifications comparison of i) the GRS-interaction-training approach (red lines; one half of the data used as training data and the other half as test data), ii) the GRS-marginal-internal approach (blue lines) and iii) lasso logistic regression (black solid lines).

**
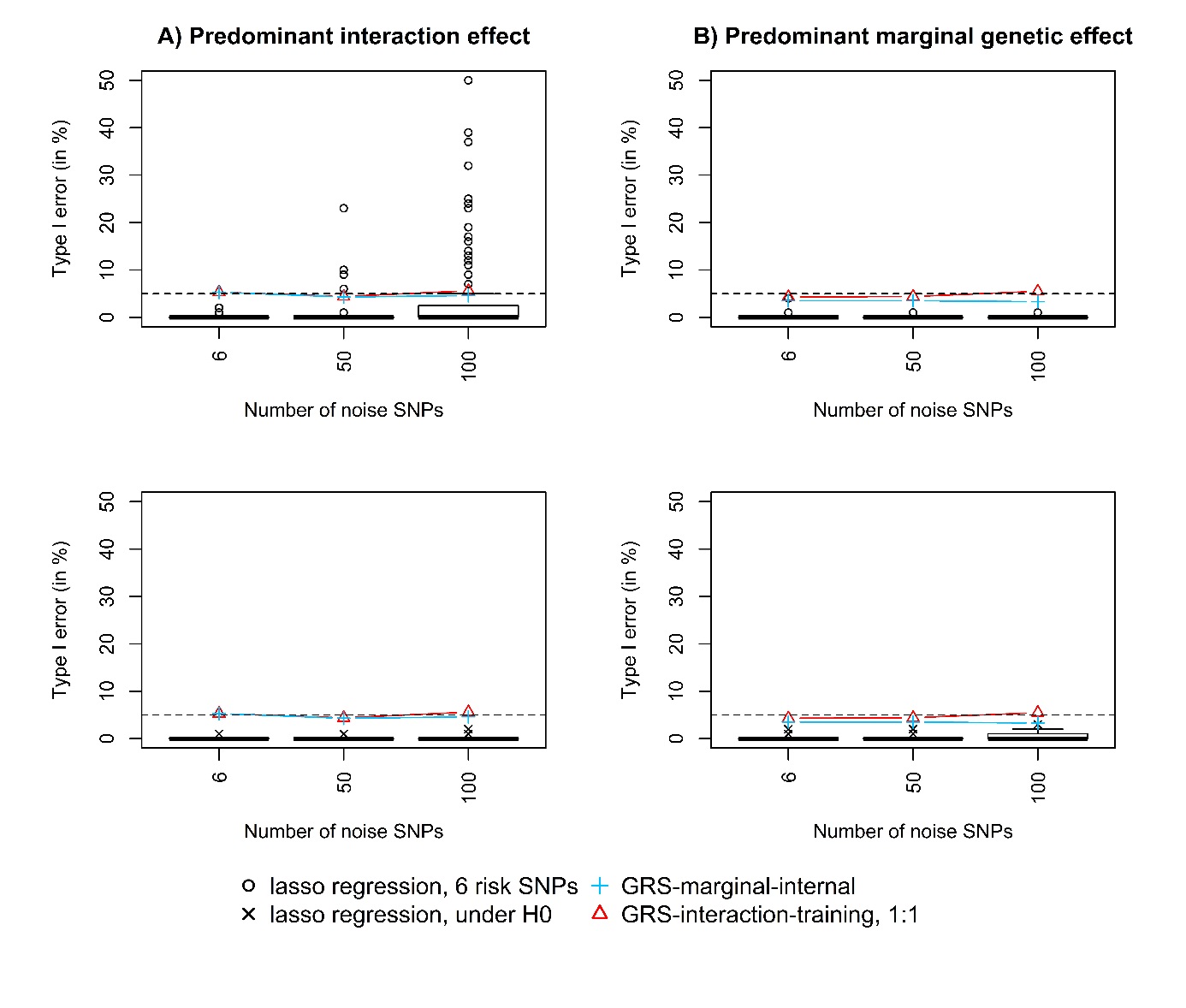
**

**Figure S2:** GRS-approaches vs. lasso regression with increasing number of noise SNPs (up to 100). Type I error comparison of i) the GRS-interaction-training approach (red lines; one half of the data used as training data and the other half as test data), ii) the GRS-marginal-internal approach (blue lines) and iii) lasso logistic regression (black boxplots). The type I error of the lasso regression was calculated as the number of false-positive interactions that were identified within the noise SNPs in the scenarios with six risk SNPs (first row, boxplots marked with “o”) as well as under the null hypothesis (no interaction present) (second row, boxplots marked with “x”) across100 replications.

**References**

1. Lockhart R, Taylor J, Tibshirani RJ, Tibshirani R. A significance test for the lasso. Ann. Stat. 2014;42:413–68.

2. Efron B, Hastie T, Johnstone I, Tibshirani R. Least Angle Regression. 2004;32:407–99.

3. Hüls A, Ickstadt K, Schikowski T, Krämer U. Detection of gene-environment interactions in the presence of linkage disequilibrium and noise by using genetic risk scores with internal weights from elastic net regression. BMC Genet. 2017;18:55.
